# Digitising Medical Nutrition Therapy in Gestational Diabetes: A Multisite Pilot Study

**DOI:** 10.1155/nrp/4193919

**Published:** 2025-12-14

**Authors:** Anna Roesler, Amy Myles, Marlien Varnfield, Kaley Butten

**Affiliations:** ^1^ Australian e-Health Research Centre, Health & Biosecurity, CSIRO, Herston, 4029, Queensland, Australia, csiro.au; ^2^ School of Psychology, Faculty of Health and Medical Sciences, The University of Adelaide, Adelaide, Australia, adelaide.edu.au

**Keywords:** food diaries, gestational diabetes mellitus, health personnel, implementation science, mobile health, participatory research

## Abstract

**Aims:**

Medical nutrition therapy, which incorporates dietary tracking, is routinely used in the management of gestational diabetes mellitus (GDM). However, paper‐based tracking approaches are associated with several challenges. Based on user feedback, the M♡THer app, an existing digital platform for GDM management, was enhanced with a digital diet diary feature. This study aimed to evaluate the uptake, usage, usability and acceptability of the diet diary feature.

**Methods:**

The diary was codesigned and trialled in three hospital services. Data collection occurred over 6 months. User engagement metrics, surveys and interviews with healthcare providers (HCPs) and app users were analysed using thematic analysis and triangulation.

**Results:**

Of the 305 M♡THer app users, 95 used the diet diary with uptake varying from 13% to 53%. Users logged their diet for an average of 33.5 days. The diary improved patient–provider communication, reducing consultation time and supporting dietary literacy. Barriers to uptake and optimal engagement included the timing of the diary introduction and usability issues.

**Conclusion:**

This study contributes to the growing evidence on digitally supported GDM care, highlighting the feasibility of applying a digital diet diary for individuals with GDM and their HCPs. The approach positively enhanced diet tracking and consultation efficiency. Future iterations should prioritise early introduction, a condensed user interface and comprehensive food library.

**Trial Registration:**

Therapeutic Goods Administration (TGA): CT‐2020‐CTN‐01167‐1‐v8

## 1. Introduction

Effective management​ of gestational diabetes mellitus (GDM) involves monitoring and stabilisation of maternal blood glucose levels (BGLs). Upon diagnosis, women are instructed to test their BGLs daily using a glucometer and share these readings with their healthcare team. Based on these data, healthcare providers (HCPs) recommend lifestyle modifications and, when necessary, pharmacological interventions. A key priority of managing GDM is educating individuals on how to optimise their dietary intake and eating behaviours, to stabilise BGLs, commonly known as medical nutrition therapy (MNT). In addition to BGL monitoring, individuals are advised to track their dietary intake using food diaries, which helps increase awareness of the relationship between diet and BGLs [1]. However, dietary tracking is associated with several challenges including underreporting of food intake [2], time constraints, recall bias and the legibility and literacy issues associated with (mostly) handwritten diaries [1].

**Figure 1:**
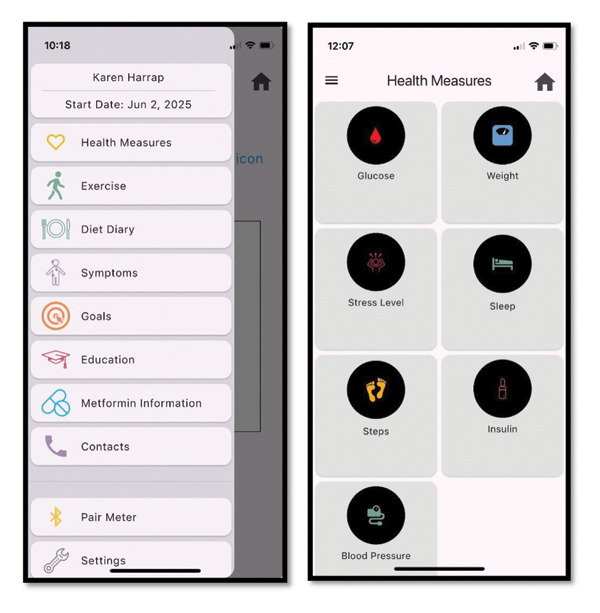
M♡THer App home menu and health measures page.

In recent years, HCPs have increasingly turned to mobile health technologies, such as smartphone applications, to assist in monitoring BGL data and, to a lesser extent, dietary intake [3]. In the management of GDM, one digital innovation is the ‘M♡THer’ platform [4]. The feasibility and acceptability of the platform have been reported previously, providing a detailed overview of its design and early evaluation [4]. Briefly, the platform consists of a mobile app (Figure 1) and HCP‐facing web‐based dashboard. Individuals can download the app onto their phone and input their relevant GDM data, including BGLs. The app allows individuals to provide a written comment on their BGL reading, which may be dietary in nature. The dashboard is accessed by HCPs to view recordings, allowing HCPs to monitor their patients’ health information remotely.

The M♡THer platform is currently being evaluated in a large‐scale, long‐term implementation study across five Queensland hospitals with the feasibility study already published [4]. As part of the ‘process and impact’ evaluation, users have indicated an interest in additional features to the platform, including a diet diary [5].

**Figure 2:**
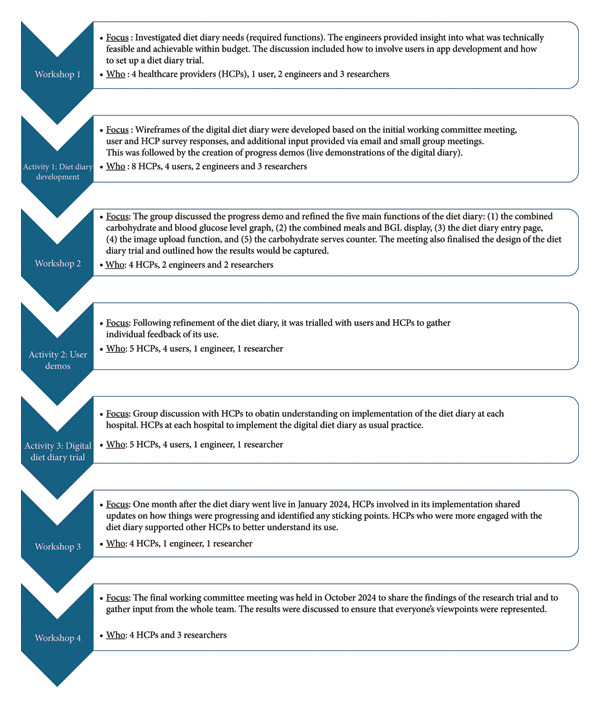
Codesign process of participatory action research to develop and trial the M♡THer platform.

**Figure 3:**
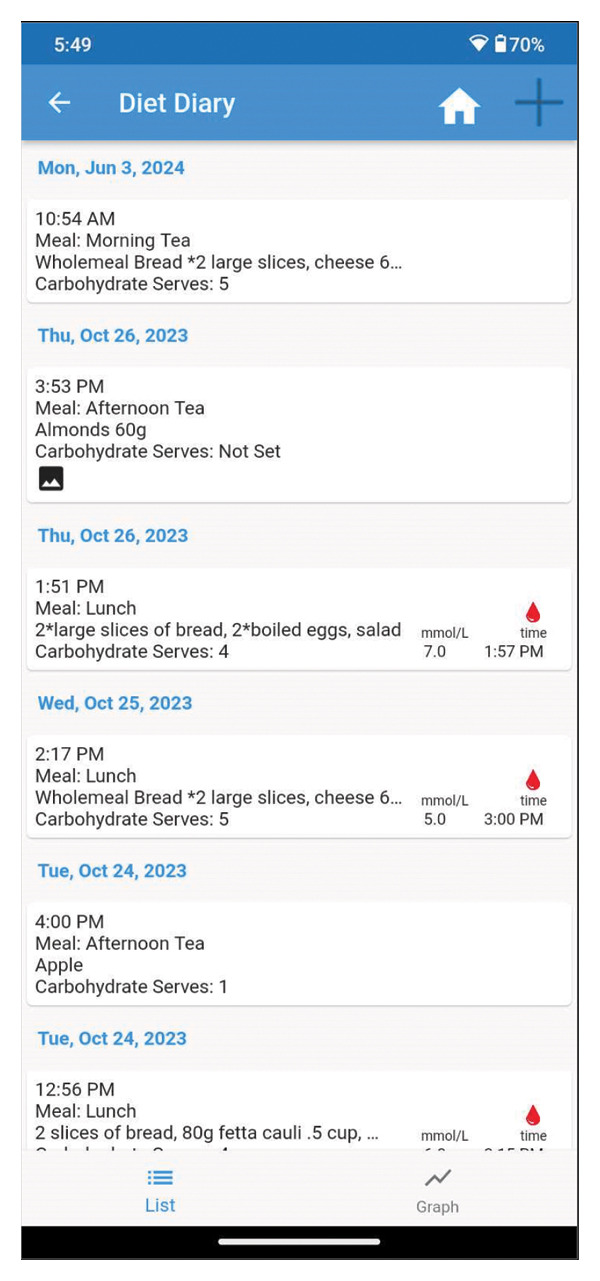
Overview page of the diet diary feature.

Informed by user feedback and utilising a participatory action research (PAR) process (Figure 2), we cocreated a novel diet diary feature (Figure 3) for the M♡THer platform. The aim of this study was to pilot test the diary, evaluating the uptake, usage, usability and acceptability of the new feature. This study took place in a real‐world setting, where each of the three hospitals involved follows its own model of care.

## 2. Methods

The development and trial of the diet diary was grounded in PAR principles, emphasising collaborative inquiry with users as coresearchers [6]. Supported by social constructivist theory [7], this approach involved users in codesigning and refining the study’s focus. Firstly, a working committee was established to guide the research process and activities (Figure 2) and consisted of HCPs using the M♡THer platform (*N* = 8), M♡THer app users (*N* = 9), and engineers and researchers (*N* = 5). Initially, only HCPs from two hospitals were involved in the PAR; however, the third hospital joined for the diet diary trial (Figure 2).

Through the PAR process, the diet diary (Figure 3) was developed and included three main components: 1) an overview page, which links dietary intake with BGL recordings; 2) an input page, designed for users to log their meals, calculate carbohydrate intake (carbohydrate counting) using a carbohydrate calculator and upload food photographs; and 3) a graph page, which provides a visual representation of BGLs and carbohydrate consumption over time.

The introduction of the diet diary to M♡THer users at the three trial sites varied. Site 1 (Metropolitan) involved four HCPs in the PAR process and familiarisation with clinical workflow. At this site, all individuals were introduced to the M♡THer app (and the diet diary) at GDM diagnosis. Individuals with GDM were encouraged to use the diet diary before their first dietitian consultation. At Site 2 (Metropolitan), two HCPs were involved in the PAR process and familiarisation with clinical workflow, similar to Site 1. Due to the site’s model of care, only individuals requiring insulin (approximately 30% of patients with GDM) were placed on the M♡THer app. Consequently, individuals with GDM were only exposed to the diet diary later in their GDM management journey (MNT is typically used as the first management approach before pharmacotherapy). While at Site 3 (Regional), HCPs at this site were not involved in the initial PAR process, nor did they complete a diet diary familiarisation session. M♡THer users were also not surveyed. The diet diary was released in a software update, and users at the site adopted the feature of their own volition. HCPs augmented their model of care to introduce the diet diary in their group education sessions at GDM diagnosis.

Informed by the technology acceptance model (TAM) [8], a commonly used framework for understanding user acceptance of technology, we aimed to evaluate uptake, usage, usability and acceptability of the diet diary feature. Uptake was determined by any interaction with the diet diary; usage was determined by the number of days the diary was used and features utilised; usability was determined by the engagement with features and user feedback; and acceptability was determined by user feedback.

Data from the M♡THer app users were collected via three methods. Firstly, user engagement metrics were analysed, including age, foods entered, carbohydrate counting completed and images uploaded. Data were collected from March to August 2024, covering a 6‐month period and including only participants who started and completed the M♡THer app within that timeframe. The second data collection method involved a 23‐question survey conducted four weeks after users began using the app, assessing their experiences. Users of the M♡THer app at Sites 1 and 2 completed this survey. Additionally, users who participated in the survey were invited to take part in an online interview to further explore the survey questions. The interviews were conducted by researchers AR and AM (AR, an experienced qualitative researcher, and AM, a PhD candidate). After participants provided consent, the interviews were conducted and recorded via Microsoft Teams.

HCPs involved in using the M♡THer platform were also invited by email to participate in semistructured online interviews to share their experiences with the diet diary. Recruitment continued until data saturation was reached, meaning no new themes or concepts emerged from additional interviews. The interviews explored experiences with the diet diary and lasted 15–60 min.

The study adheres to the Consolidated Criteria for Reporting Qualitative Research (COREQ) [9]. Triangulation was employed to integrate insights from these different data sources and to crossverify results, enhancing the validity and reliability of the conclusions drawn.

For analysis of the data, descriptive statistics were applied to analyse quantitative survey responses. Free‐text survey responses were analysed inductively, while interview transcripts were examined using the framework method as described by Ritchie and Lewis [10]. This approach emphasises systematic coding, framework development and interpretive consensus rather than statistical measures of reliability, such as Kappa. Interviews were transcribed using Microsoft Teams and edited verbatim. Two researchers (AR & AM) independently familiarised themselves with the first three transcripts, generated initial codes and then met to compare and consolidate a preliminary analytical framework. Discrepancies were resolved through discussion, with consensus guiding refinement of the framework. This coding framework was subsequently applied to the remaining transcripts by AR, with ongoing consultation between coders to ensure coherence and interpretive depth. Data were then charted into a case‐by‐code matrix to support theme development and comparison across participants. Final themes and illustrative quotes were reviewed and refined collaboratively by all authors. To further enhance the credibility and trustworthiness of the findings, the collated results were shared with the PAR working committee, whose feedback informed the final interpretation.

Recruitment of participants was conducted in accordance with the Declaration of Helsinki (1964), and all participants provided informed consent before participation. Ethics approval for the collection of user engagement metrics and surveys related to the M♡THer platform was granted by the Metro South Human Research Ethics Committee (HREC/2019/QMS/56607). Additionally, ethics approval for the interviews conducted as part of the trial was obtained under HREC/2022/QMS/84491. The study adheres to the COREQ checklist (see Supporting File 1).

## 3. Results

The following results represent findings from three data sources: 1) app user engagement metrics, 2) interviews with HCPs and M♡THer app users and 3) survey responses (Table 1). The data are integrated to provide a comprehensive overview of user experiences, including uptake, usage, usability and acceptability of the diet diary.

**Table 1:** Demographic details and feedback from survey respondents using the M♡THer app.

Survey question	Response category	%	(*n*)
Hospital	Site 1	65	(11)
	Site 2	35	(6)
Age group	23–27	12	(2)
	28–32	29	(5)
	33–37	29	(5)
	38 +	29	(5)
Previously had GDM	Yes	47	(8)
	No	23	(4)
	N/A	29	(5)
Born in Australia	Yes	71	(12)
	No	29	(5)
Education	Completed high school	6	(1)
	TAFE certificate, diploma or trade certificate	47	(8)
	A degree	47	(8)
Management of GDM	Diet/exercise	71	(12)
	Metformin	59	(10)
	Insulin	59	(10)
*Uptake*			
I used the diet diary	Yes	35	(6)
	No	59	(10)
	Other	6	(1)
*Usage*			
I viewed the carbohydrate and BGL graph	Never	1	
	Sometimes	4	
	Often	2	
I recorded my carbohydrate portions	With the carbohydrate calculator	2	
	By tallying myself	1	
	No	4	
*Usability*			
I was provided with instructions on how to use the diet diary	Yes	59	(10)
	No	41	(7)
I had enough information to use the diet diary	Enough	77	(13)
	Not enough	23	(4)
The diet diary was complex	Agree	29	(5)
	Disagree	24	(4)
	Neutral	47	(8)
*Acceptability*			
The diet diary was useful for understanding carbohydrate intake	Agree	24	(4)
	Disagree	29	(5)
	Neutral	47	(8)
Using the diet diary was a positive experience	Agree	35	(6)
	Disagree	6	(1)
	Neutral	59	(10)
The diet diary helped with blood glucose management	Agree	29	(5)
	Disagree	29	(5)
	Neutral	41	(7)

App User Uptake and Engagement Metrics: Across the three hospitals, 95 M♡THer app users adopted the diet diary of the 305 M♡THer app users. Uptake patterns closely reflected how the diet diary was introduced. Site 1, where the diary was introduced consistently at diagnosis and before dietitian review, recorded the highest uptake (53%). Site 2, where introduction occurred later and only for individuals progressing to insulin therapy, had the lowest uptake (13%). Site 3, where the diary was not formally introduced but became available through a software update, achieved moderate uptake (39%). These findings suggest that both the timing of introduction and integration into existing workflows are critical drivers of adoption. Logging within the diet diary varied between users with 80% logging between 2 and 130 days (33.5 days mean [SD32.81, *N* = 94]) across all sites. Approximately a quarter of diet diary users added photographs of the food that they ate (Site 1, 27.91%; Site 2, 33.33%; and Site 3, 26.47%)

Across sites, 31% of users included carbohydrate counting in their entries. When carbohydrate counting was checked for accuracy, discrepancies often occurred when foods were misidentified, particularly for less common items, such as buckwheat (incorrectly listed as baked beans) or rice paper (listed as pasta). Other errors included categorising ‘high in added sugars’ food versions, such as sugary cereal (listed as high‐fibre cereal), or misclassifying discretionary foods, such as chips (listed as mashed potatoes), and pies (listed as bread). Additionally, some foods, such as chocolate, honey and certain drinks (e.g. coconut water), were often omitted from carbohydrate counting altogether.

Interviews and Survey: Of 216 M♡THer app users at Sites 1 and 2 (noting Site 3 did not receive surveys), 17 users responded to the survey conducted 4 weeks after starting the M♡THer app, yielding an 8% response rate. Demographic details and responses are summarised in Table 1.

A total of 12 HCPs across three sites participated in interviews (10 additional HCPs were invited; 9 did not respond and 1 recommended someone else), representing five dietitians, two antenatal care providers and five diabetes educators. Three individuals with GDM (app users) were interviewed (1 additional individual was invited, but did not respond). The themes are categorised under two main headings: (1) barriers to uptake and (2) benefits of the diet diary.

User interface was the first barrier to uptake of the diet diary. Before the introduction of the diet diary, HCPs often advised M♡THer app users to record dietary information in the BGL comment section of the app when entering their BGLs. Some HCPs continued to recommend this approach, as they found it more convenient for reviewing patient data.

‘I find sometimes it′s more helpful for us if ladies enter their diet in the notes section of the blood sugar part and the reason for that is then you′re not flicking backwards and forwards between two tabs’. HCP 1

Some HCPs also felt that recording the dietary information within the BGL comments was easier for M♡THer app users.

‘It would be easier if the women didn′t have to go into multiple different sections of the app to make all of those entries if it was just a bit of a one stop sort of place’. HCP 2

This sentiment was shared by some M♡THer app users as well.

‘I used the comments section of the glucose diary to map my food, because it is one place and doesn’t take a considerable amount of time’. M♡THer app user 9

It is believed that the timing of the introduction of the M♡THer app to patients impacted the uptake of the diet diary. At Site 1, all users were enrolled on the M♡THer app following GDM diagnosis. The midwife introduced the app, including the diet diary. At this time, individuals were requested to record their diet for the dietitian to review.

‘The diabetes educators contacts the women when they′re first diagnosed, after [a midwife] has registered them, and then we just go through and tell them how to use the app, like access to the education component and then to suggest that they use the diet diary feature so that the dietitian can at least review [the diet prior to the appointment]’. HCP 3

Meanwhile, at Site 3, the diet diary was not formally introduced to the healthcare team (it was released to the app as part of a software update under ethical approval). Despite not participating in any education about the feature, the HCPs at the site began utilising the diet diary on their own initiative. The feature was introduced to women in the GDM group education session following diagnosis.

‘The model of care is the group education for everyone where they have the initial dietitian and education, and set up with a glucometer and set up with the app…. I′ll briefly talk about the diet diary part. So the three parts that I kind of highlight to ladies is the blood sugars, the diet diary and the insulin, because they′re the most helpful things to enter for us to advise women remotely on’. HCP 1

At Site 2, because of the site service delivery model, only individuals who progress to insulin management go onto the M♡THer app and could therefore access the diet diary.

‘And due to the large number of ladies with GDM at this site, it meant that not all ladies could go onto the app initially… There′s roughly 1000 women who experience GDM here a year, so we just cannot look at all of those people all the time … [Participants] First diagnosed with GDM have their first education session. They′re given a week′s [paper] diary that they put their first week of blood sugar levels and what they eat, and then that gets shown to the dietitian at their first appointment, which is about a week or two after their education. After that week, I believe they don′t have to record anything… they just see the dietitians every two to four weeks and talk about what they′re eating then’. HCP 7

It was shared by some of the HCPs at Site 2 that the diet diary was not as much of a benefit after the initial GDM diagnosis period.

‘There′s no point [in filling in the diet diary] after they go on insulin… I don′t ask them for a food diary again. And dietitians’ kind of ask them what have you been eating, but they never formally ask them to write down a food diary’. HCP 8

‘My job isn′t specifically to review their diet, so I guess I don′t totally focus on the diet diary, but I do find it really, really good as a resource when their levels are high. Otherwise, I just look after eighty of them [people with a GDM diagnosis] and I review them twice a week, so I don′t have time to read what they′re eating unless there′s a problem’. HCP 8

However, some HCPs championed the diet diary’s use and encouraged positive perceptions.

‘I tell them that it′s [the diet diary] there. It′s not compulsory, but if they would like to use it, they can…I preface it by saying hey, this will give us a really good idea of what you′re eating and how much and when you′re eating and it can help the dietitian give you some really good advice’. HCP 5

Further, HCPs who were in touch with other HCPs could help troubleshoot and overcome barriers to the diet diary feature use.

‘At first I was a bit confused. When I click on diet diary and I just saw the blood sugars. I was a bit confused about why the diary wasn′t there, but [the dietitian] kind of explained to me that you just hover over the meal, which kind of makes sense now’. HCP 8

Benefits of the diet diary were also highlighted. The first of these included improved patient–provider communication and efficiencies. Of the HCPs, dietitians unanimously indicated that the diet diary was extremely useful for them. The dietitians appreciated that the feature allowed them to access the dietary information of patients before a consultation. In the consultation, the dietitian did not have to spend their time gathering dietary information; instead, they could clarify and focus on providing personalised education and support.

‘If they′re using the app, and they′re coming in with that data already there, then we′ve got that to utilise. We′re not doing their diet histories and that sort of stuff with people. We can just do targeted exact education, which is what this group needs’. HCP 9

Furthermore, having the diet diary filled in along with BGLs provided a more complete picture to what was happening with the individual, allowing for a more tailored conversation.

‘I find it [the diet diary] really helpful to give me a good background of what′s going on with the ladies, because I′ve got the whole picture in front of me. I′ve got their blood glucose, I′ve got their diet, I′ve got their medical history from the chart, and I can usually have a very productive conversation with the ladies… I can give them really practical advice’. HCP 6

Convenience was also a benefit. The dietitians indicated that the digital diet diary was taken up and filled in by more individuals with GDM than the paper diary.

‘I have had people comment that the app is convenient and easy, by women who have used the paper system in the past …and now they have come into a subsequent pregnancy and use the app, they have commented about how it′s easier and more convenient’. HCP 1

HCPs also shared that the added benefit of the diet diary is the reduction of cognitive load for users in remembering to bring the paper diary with them to appointments. It also had implications for personal and structural resources, by allowing for more flexibility in monitoring.

‘The other thing is, paper‐based is reliant on the woman completing it and filling it in, and bringing it with them. A lot of ladies will want to, they just won′t bring them’. HCP 5

‘It just makes it a bit easier for us and for the women because it means we can review people outside of the clinics and more easily and we can save women [attending] some appointments’. HCP 2

Users indicated that the diet diary went beyond improving their communication with the HCP and saving time, and it also helped them visualise their data and providing them with insight, providing them with confidence in their dietary choices.

## 4. Discussion

This study evaluated the uptake, usage, usability and acceptability of a digital diet diary within a mobile health app for managing GDM. Understanding the demand for digital solutions in GDM care is crucial as health systems evolve and invest in technology.

The diet diary was well accepted, with up to 53% engagement at GDM diagnosis. The contrast in uptake across sites highlights the importance of implementation strategy. Early introduction at diagnosis appears to normalise diary use, while later or selective introduction reduces relevance for many users. Site 3’s moderate uptake, despite minimal formal promotion, indicates that accessibility alone can generate engagement, though not necessarily at consistent levels. Defining optimal uptake of a GDM diet diary is challenging due to the variability of MNT approaches; research suggests fewer than a third of Queensland hospitals offer the minimum schedule of dietetic appointments [11]. No Australian clinical practice guidelines dictate diet tracking frequency [12], yet MNT remains the primary treatment [13]. Barnes et al. [14] suggest changes in diagnostic thresholds are responsible for more women being diagnosed and subsequently ‘milder degrees of GDM’, of which MNT is used as the primary management approach. This circumstance is reflected in our study environment, whereby Site 2 only utilised the digital support (M♡THer) for individuals on insulin because they had insufficient resources to onboard such a large cohort using MNT. Interestingly, once the trial was complete, the site augmented their service delivery model and started providing the M♡THer platform to all individuals with GDM, no matter their management type (i.e. MNT or pharmacotherapy).

In a similar vein, but less expected, was the uptake of the diet diary by Site 3 who had not been involved in the initial PAR process but received the revised version of the platform as a consequence of the software update. Of the 88 individuals who had access to the app, 34 (39%) utilised the diary. This independent adoption provided us with a relatively unbiased indication of the diary’s overall utility and acceptability. Other Australian research [15], which evaluated digital diet diaries in a clinical setting, suggests that there can be considerable errors in digital dietary recording which could pose barriers to use in clinical settings. However, the feedback we received suggests that the potential for errors is less important than the opportunity the digital diary provides for dialogue and education.

Indeed, our prior research [5, 16, 17] and that of others [18] suggest there is a strong desire by individuals with GDM to work more collaboratively with their HCPs and feel a sense of agency regarding their management. This study contributes further evidence of how digital approaches can assist in that process. Specifically, the benefits of digital data capture (particularly with photographs) and how it can act as a conversation tool.

Individuals in this study tracked their dietary intake for an average of 1 month. This is longer than the typical 3‐day to 1‐week period recommended by dietitians at the study sites. This extended tracking may be due to the accountability it provided, helping individuals stay mindful of the foods they consumed while gaining valuable insights [19]. Even at Site 2, where individuals were past the MNT approach to management, 13% still utilised the diary. Diet tracking can increase self‐control and confidence, as individuals gain insight into their BGLs and dietary choices [20]. It also may relate to the convenience of tracking digitally. Convenience was expressed as desired by many participants, with users indicating their interest in having a ‘one‐stop shop’ as opposed to multiple screens to toggle through. In their evaluation of a digitised 24‐h recall diary tool, Gianfrancesco et al. [21] also found that participants desired seeing their BGLs and diet in one location. Future iterations should consider further condensing the interface.

Overall, this study benefitted from a pragmatic approach which included sites at varying levels of technology readiness, which in turn facilitated a ‘real‐world’ adoption. The PAR process ensured that the diet diary was codesigned with both HCPs and individuals with GDM, enhancing relevance and usability. Triangulation across app engagement metrics, surveys and HCP interviews also provided complementary insights that strengthen the credibility of the findings. However, there are several limitations to be acknowledged. The small sample sizes, particularly the limited user interviews (*n* = 3) and exclusion of Site 3 from survey data collection, restrict the representativeness of findings, and conclusions should be considered exploratory. While our survey captured some demographic characteristics (e.g. age, education, prior GDM history and country of birth; Table 1), we were unable to collect more detailed sociodemographic and health information (e.g. parity, specific ethnicity or detailed GDM history). This limited our ability to examine how these factors may have shaped diet diary uptake and engagement. Given the established role of ethnicity and cultural background in GDM risk and care experiences [22], future research should prioritise capturing these data to inform equitable and culturally sensitive digital interventions. Moreover, the lack of a comparison with paper diary use restricts insight into relative preferences for digital versus traditional approaches. A direct comparison between digital and traditional models of diet tracking is recommended. It will also be important to investigate how HCP support, and particularly the presence of local champions who advocate for digital tools, influences user engagement and the successful integration of such systems into routine care. Taken together, these strengths and limitations highlight the need for cautious interpretation, while also underscoring the practical value of our findings for informing future iterations of digital support in GDM care.

## 5. Conclusion

This study demonstrates the feasibility and acceptability of a digital diet diary integrated within the M♡THer platform for GDM management. The diary supported extended tracking, strengthened patient–provider communication and showed how implementation strategies, such as timing and workflow integration, shape uptake. While further work is needed to optimise design and evaluate the diary against traditional approaches, these findings provide an important foundation for advancing digitally supported MNT in maternity care.

## Disclosure

All authors approved the final version.

## Conflicts of Interest

The authors declare no conflicts of interest.

## Author Contributions

K.B. and A.R. conceptualised the study. A.R. and A.M. collected and analysed the data. All authors reviewed the final themes. A.R. and K.B. drafted the manuscript, and all authors contributed to editing.

## Funding

The authors received no specific funding for this work.

## Supporting Information

Supporting File 1. COREQ Checklist: This file provides the completed COREQ checklist outlining the reporting standards met in this study.

## Supporting information


**Supporting Information** Additional supporting information can be found online in the Supporting Information section.

## Data Availability

De‐identified survey and app engagement data that support the findings of this study are available from the corresponding author upon reasonable request. Interview transcripts cannot be shared due to confidentiality restrictions and the conditions of ethics approval.
